# Dental Calculus Links Statistically to Angina Pectoris: 26-Year Observational Study

**DOI:** 10.1371/journal.pone.0157797

**Published:** 2016-06-23

**Authors:** Birgitta Söder, Jukka H. Meurman, Per-Östen Söder

**Affiliations:** 1 Department of Dental Medicine, Karolinska Institutet, Huddinge, Sweden; 2 Department of Oral and Maxillofacial Diseases, University of Helsinki and Helsinki University Hospital, Helsinki, Finland; University of Bologna, ITALY

## Abstract

**Objectives:**

Dental infections, such as periodontitis, associate with atherosclerosis and its complications. We studied a cohort followed-up since 1985 for incidence of angina pectoris with the hypothesis that calculus accumulation, proxy for poor oral hygiene, links to this symptom.

**Methods:**

In our Swedish prospective cohort study of 1676 randomly selected subjects followed-up for 26 years. In 1985 all subjects underwent clinical oral examination and answered a questionnaire assessing background variables such as socio-economic status and pack-years of smoking. By using data from the Center of Epidemiology, Swedish National Board of Health and Welfare, Sweden we analyzed the association of oral health parameters with the prevalence of in-hospital verified angina pectoris classified according to the WHO International Classification of Diseases, using descriptive statistics and logistic regression analysis.

**Results:**

Of the 1676 subjects, 51 (28 women/23 men) had been diagnosed with angina pectoris at a mean age of 59.8 ± 2.9 years. No difference was observed in age and gender between patients with angina pectoris and subjects without. Neither was there any difference in education level and smoking habits (in pack years), Gingival index and Plaque index between the groups. Angina pectoris patients had significantly more often their first maxillary molar tooth extracted (d. 16) than the other subjects (*p* = 0.02). Patients also showed significantly higher dental calculus index values than the subjects without angina pectoris (*p* = 0.01). Multiple regression analysis showed odds ratio 2.21 (95% confidence interval 1.17–4.17) in the association between high calculus index and angina pectoris (*p* = 0.015).

**Conclusion:**

Our study hypothesis was confirmed by showing for the first time that high dental calculus score indeed associated with the incidence of angina pectoris in this cohort study.

## Introduction

The link between chronic oral infections and atherosclerosis and its complications, such as heart infarction and stroke, has been explored in numerous studies.[1–3] Periodontal diseases, in particular, have been intensively investigated in this perspective.[4] A recent study from Spain showed that periodontitis was associated with acute myocardial infarct size as measured by serum troponin I and myoglobin levels, thus further strengthening the association.[5]

A dental biofilm is formed on the non-shedding oral surfaces, namely, on teeth, dental restorations, and prostheses. The biofilm may absorb calcium or phosphate ions from saliva and gingival crevicular fluid resulting in dental calculus [6]. Hence, dental calculus is primarily composed of calcium phosphate mineral salts covered by non-mineralized bacterial biofilm. Although the first evidence of calcification in the biofilm is seen after only a few days, mature calculus requires months or even years to develop.[7,8] Lactate dehydrogenase and alkaline and acid phosphatase activities have been detected in dental plaque suggesting an enhanced calcification by the plaque enzymes.[9] Viable aerobic and anaerobic bacteria have been detected in supragingival calculus while subgingival calculus provides an excellent environment for further microbial adhesion and growth.[10] Periopathogens such as *Aggregatibacter actinomycetemcomitans*, *Porphyromonas gingivalis*, and *Treponema denticola* have been found within the deep recesses of the structural channels and lacunae of both supra- and subgingival calculus.[11,12] Bacteria are not essential for calculus formation, but they facilitate its development. The average microscopic count of bacteria in non-mineralized dental plaque has been calculated to be up to 2.1 ×10^8^/mg wet weight.[13, 14] Oral microbiota contains hundreds of species. [15,16]

Hence, high amount of calculus indicates that oral hygiene has been poor for months or even years.[17] Subsequently, a high dental calculus index score might indicate a long-lasting oral infection burden which causes up-regulation of systemic inflammatory reactions.

Accumulation of dental calculus is a clear risk for the development of periodontitis and there is consensus among professionals that removing plaque and calculus mechanically prevents the disease.[18] We have observed in our cohort study from Sweden that high dental calculus index score associated with 2.3 times the odds ratio for cardiac death.[19] From the same cohort, periodontitis was shown to link with carotid artery intima media thickness and thus presented risk for stroke. [20] In another case-control study from our group investigating patients with angiographically verified coronary artery disease we found that oral health status and oral health behaviour was worse among the patients than in cardiologically healthy controls.[21] Indeed, the statistical association between poor oral health and atherosclerosis is well established since many years, and treatment of periodontitis has been shown to improve the situation. [22,23] However, overall scientific evidence is still weak in this regard.[24]

Chest pain or angina pectoris (AP) is a symptom of coronary artery disease and prodromal sign for heart infarction. Angina is in practice caused by coronary artery atherosclerosis and manifests as acute coronary syndrome. A review in the Nature recently stated that the clinical entities of acute coronary syndrome have been important contributors to global morbidity and mortality for at least 250 years.[25] Hence, we here discuss a significant clinical problem.

In the present study we analyzed the prevalence of AP from our Swedish cohort that was followed-up for 26 years. The association between dental calculus index score and angina was analyzed in the oral infection—atherosclerosis paradigm. We hypothesized that high dental calculus index, a marker of poor oral hygiene and potential risk for periodontitis, links with registered AP symptoms.

## Material and Methods

**Fig 1:**
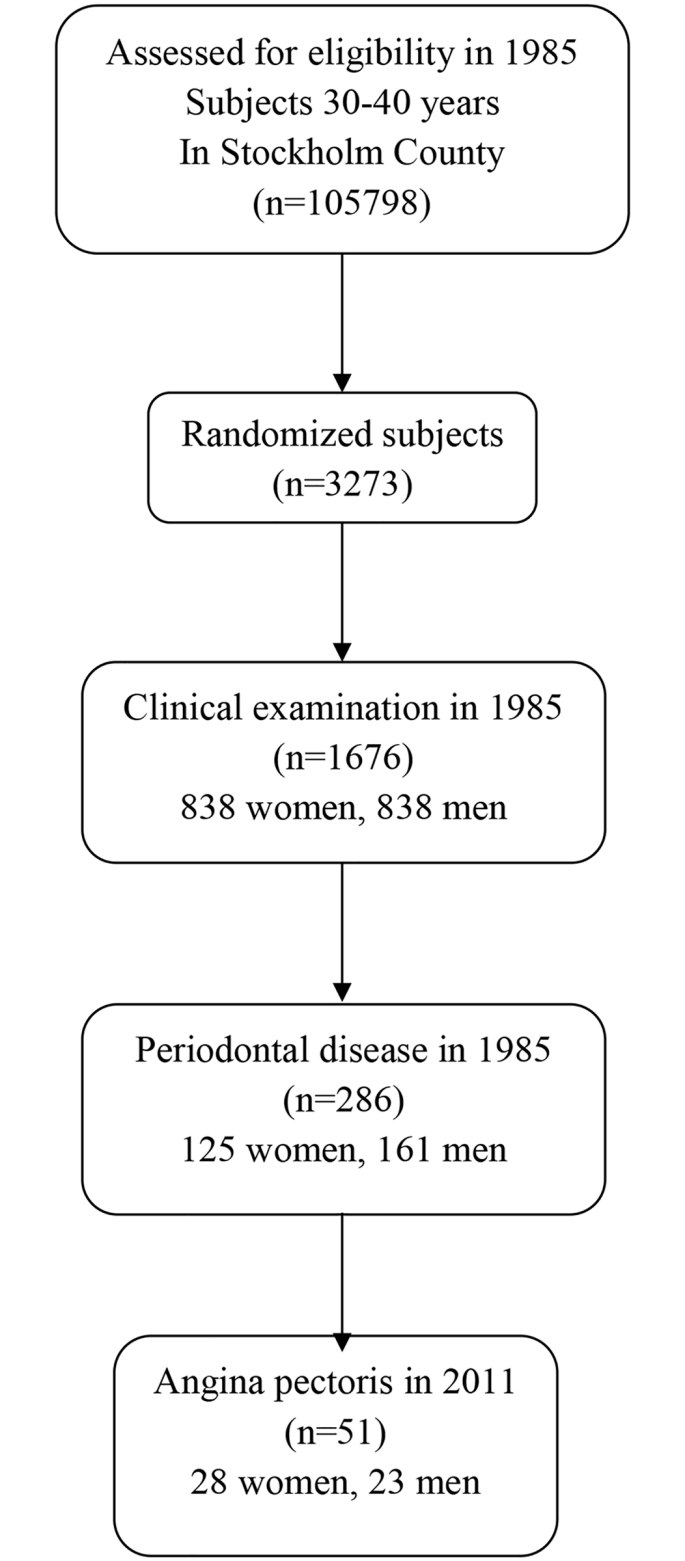
Study profile.

Our cohort of 1676 subjects has been described in detail by Söder et al. (2007) and the study profile is shown in Fig 1.[26] The participants were invited by a letter. Those accepting to participate gave their consent also regarding use of clinical data. The patient records/data were anonymized and de-identified prior to analyses. In brief, the national hospital records of Sweden were used to analyze the association of baseline oral health parameters with respect to angina pectoris. For statistics, analysis of variance, chi-square tests, multiple regression analysis, and multiple logistic regression analysis were used as appropriate. Multiple logistic regression analysis was used to compare the incidence of angina pectoris, according to the state of oral health at baseline, while simultaneously controlling for several potential confounding variables. We included in the statistical model age, gender, periodontal disease, dental plaque index (PLI),[27] gingival inflammation index (GI),[28] and calculus index (CLI).[29] socioeconomic status, working, and smoking habits. Smoking habits were dichotomized into number of smokers (ever smokers) and number of never smokers. Smoking was calculated as pack-years of smoking (number of cigarettes per day multiplied by 365 days and divided by 20 [number of cigarettes in a pack] = the number of packages per year multiplied by the number of years smoked). The model with the confounders was correlated to the occurrence of angina pectoris in the hospital patient register. A backwards elimination method was used to control for multicollinearity (correlation between confounders). The statistical model was tested according to Cox & Snell and Nagelkerke.

Differences between data sets with *p* < 0.05 were regarded as significant. All *p*-values are two-tailed, and confidence intervals were calculated at the 95% level. All statistical analyses were performed using the spss version 14.0 (Chicago, IL, USA).

The study was approved by the Ethics Committee of the Karolinska Institutet and Huddinge University Hospital in Sweden (Dnr 101/85 and revised in 2012/590-32). The study is in accordance with the Declaration of Helsinki.

### Angina pectoris symptoms and socioeconomic data

Data about in hospital verified AP symptoms were obtained from the Center of Epidemiology, Swedish National Board of Health and Welfare, Sweden. The diagnoses for AP had been given to subjects between 55 to 64 years of age. The data were classified according to the WHO International Statistical Classification of Diseases and Related Health Problems (ICD-9 and ICD-10). Socioeconomic data were further obtained from the National Statistics Centre, Örebro, Sweden. The data for angina pectoris symptoms in 2011 were compared with the clinical data from 1985. In brief, data from the Center of Epidemiology, Sweden were used to analyze the association of baseline oral health parameters with respect to AP.

### Statistical analysis

For statistics, analysis of variance, chi-square tests, and multiple logistic regression analysis were used as appropriate. Multiple logistic regression analysis was used to compare the incidence of AP, according to the state of oral health at baseline, while simultaneously controlling for several potential confounding variables. We included in the statistical model age, gender, socioeconomic status, working and smoking habits. Smoking habits were dichotomized into number of smokers (ever smokers) and number of never smokers. Smoking was calculated as pack-years of smoking (number of cigarettes per day multiplied by 365 days and divided by 20 [number of cigarettes in a pack] = the number of packages per year multiplied by the number of years smoked). The model with the confounders was correlated to the occurrence of AP in the hospital patient register. A backwards elimination method was used to control for multicollinearity (correlation between confounders). The statistical model was tested according to Cox & Snell and Nagelkerke. Differences between data sets with *p* < 0.05 were regarded as significant. All *p*-values are two-tailed, and confidence intervals were calculated at the 95% level. All statistical analyses were performed using the spss version 21.0 (Chicago, IL, USA).

### Ethical considerations

The study was approved by the Ethics Committee of the Karolinska Institutet and Huddinge University Hospital in Sweden (Dnr 101/85 and revised in 2012/590-32). The study is in accordance with the Declaration of Helsinki. The participants were invited by a letter. Those accepting to participate gave their consent also regarding use of clinical data. The patient records/data were anonymized and de-identified prior to analyses.

## Results

Of the 1676 subjects, 51 (28 women and 23 men) had been diagnosed with AP with a mean age of 59.8 ± 2.9 years during the 26 years of follow-up. Demographic and clinical oral health data at baseline in 1985 of the subjects with and without Angina pectoris by the year 2011 are given in Table 1.

**Table 1:** Demographic clinical oral health data of 1676 subjects at baseline examination 1985 with and without angina pectoris 2011.

	Angina pectoris (n = 51) Number, Mean ± SD	No Angina pectoris (n = 1625) Number, Mean ± SD	*p*
Gender, Woman/Men	28/23	810/815	NS
Age, in 2011 (years)	59.77 ± 2.90	59.7 ± 2.85	NS
Smoking, pack-year	4296.8	5365.8	NS
Education, Compulsery/Higher	3/48	184/1337	= 0.02
Income (Swedish Crowns x 1000)	1750.33	1805.82	NS
Plaque Index	0.78 ± 0.59	0.71 ± 0.49	NS
Gingival inflammaion (GI)	1.36 ± 0.61	1.27 ± 0.53	NS
Calculus index	0.66 ± 0.76	0.45 ± 0.58	= 0.01
Missing first maxillary molar (d.16)	0.12 ± 0.32	0.47 ± 0	= 0.02
Periodontal pockets	1.33 ± 3.33	0.86 ± 2.82	NS

No difference was observed in age and gender between the patients with AP and subjects without. Neither was there any difference in smoking habits (in pack years) or income between the groups. The level education was significantly higher in the No Angina pectoris group.

No significant difference was found in gingival index score (GI), plaque index score and number of periodontal pockets between the groups.

Calculus index score was significantly higher in the AP group (*p* = 0.01). AP patients also had significantly more often their first maxillary molar tooth (d. 16) extracted than the other subjects (*p* = 0.02) Table 1. In the multiple logistic regression analysis with Angina pectoris as the dependent variable and several independent variables, namely age, gender, education, income, socioeconomic status, smoking habits, periodontal pockets, missing molars respectively, high calculus index appeared to be a principal independent predictor associated with 2.21 times the odds of angina pectoris (*p* = 0.015). The results are given in detail in Table 2 as well as in S1 Dataset.

**Table 2:** The results of multiple logistic regression analysis of the relationship between Angina pectoris as a dependent variable and several independent variables (age, gender, smoking, social status, working, periodontal disease, dental plaque, dental calculus and gingival inflammation.

Dependent Variable	Explaining Variable	β	X2	p-value	OR (95% CI)
**Angina pectoris**	**Dental Calculus**	**0,792**	**5,952**	**0.015**	**2.21(1.17–4.17)**

Cox & Snell 0,005 square, Nagelkerk 0,019 square

## Discussion

This straightforward cohort study showed for the first time an interesting statistical association between high dental calculus index scores and angina pectoris. The result thus confirmed our study hypothesis. The strengths of the study were the 26-year observation time and the homogenous Swedish population of the cohort. The limitation was that we did not have other background variables for AP risk factors, such as blood pressure values (hypertension) and lipid profiles, except those here mentioned. Nevertheless the current result might be used for further hypothesis generation regarding the causality of atherosclerosis. Namely, it was an astonishing observation indeed that only calculus index emerged as significant explanatory factor in the regression analysis conducted. However, it should be emphasized that no true causality can be discussed based on this observational study.

Starkhammar Johansson et al. (2008) reported in their case-control study on 161 patients with coronary heart disease (162 controls) that severe periodontitis was more prevalent among the patients but they did not report any calculus index scores.[30] In another case-control study on 33 patients with heart infarction or unstable angina no difference was found in oral health parameters between the groups and neither there was any calculus index recorded.[31] On the other hand, a study on 51 patients with heart infarction and 49 controls an odds ratio 5.87 (95% CI, 1.17–29.4) was found between chronic periodontitis and heart disease.[32] Periodontitis results from long-term poor oral hygiene and supposedly calculus accumulation among the patients would have been seen. These examples show the controversy in the existing literature in this topic.

Our observation that the AP patients had more often their molar teeth extracted when compared with the subjects with no AP also supports the dental infection theory. Namely, missing molars indicate earlier infections in the teeth in question, caries and/or periodontal disease.[33,34]

Dental calculus can be regarded as a depot of microbial toxic substances and other irritants.[35] In calculus the micro-organisms are encapsulated in the calcified biofilm and if not frequently removed it may indeed cause not only local problems (gingivitis and periodontitis) but also due to hematogenic spread of microbes and their products a number of systemic complications. In general, however, the association between calculus formation and systemic health has not been widely investigated. Hence our present results provide new insight into this partly controversial area. [36,37] It is clear, however, that in future studies a more comprehensive palette of angina pectoris risk factors need to be considered than was available in the present database.

## Conclusion

In conclusion, our study hypothesis was confirmed as the present findings showed for the first time a statistical association between high dental calculus index score and incidence of angina pectoris in this cohort study.

## Supporting Information

S1 Dataset The results of multiple logistic regression analysis. (SPV)
